# Effect of routine probiotic, Lactobacillus reuteri DSM 17938, use on rates of necrotizing enterocolitis in neonates with birthweight < 1000 grams: a sequential analysis

**DOI:** 10.1186/1471-2431-12-142

**Published:** 2012-09-04

**Authors:** Chelsea Hunter, Mary Ann VT Dimaguila, Peter Gal, John E Wimmer, James Laurence Ransom, Rita Q Carlos, McCrae Smith, Christie C Davanzo

**Affiliations:** 1 Women’s Hospital of Greensboro, Cone Health, 801 Green Valley Road, Greensboro, NC, USA; 2 Piedmont Neonatology, 628 Green Valley Road, Suite 210, Greensboro, NC, USA; 3 University of North Carolina, Eshelman School of Pharmacy, Chapel Hill, NC, USA; 4 Greensboro Area Health Education Center, Greensboro, NC, USA

**Keywords:** Necrotizing enterocolitis, * Lactobacillus reuteri * DSM 17938, Probiotic, Extremely low birth weight

## Abstract

**Background:**

Necrotizing enterocolitis (NEC) is a disease in neonates, often resulting in death or serious medical or neurodevelopmental complications. The rate of NEC is highest in the smallest babies and many efforts have been tried to reduce the rate of NEC. In neonates born below 1500 grams, the rate of NEC has been significantly reduced with the use of various probiotics. This study examines the impact of routine use of a probiotic, * Lactobacillus reuteri * DSM 17938 (BioGaia®), on the rate of NEC in neonates at highest risk for developing NEC, those with birth weight ≤1000 grams.

**Methods:**

This is a retrospective cohort study comparing the rates of NEC in neonates with birth weight ≤ 1000 grams. The groups are separated into those neonates born from January 2004 to June 30, 2009, before introduction of * L. reuteri *, and neonates born July 2009 through April 2011 who received routine * L. reuteri * prophylaxis. The chart review study was approved by our institutional review board and exempted from informed consent.

Neonates were excluded if they died or were transferred within the first week of life. The remainder were categorized as having no NEC, medical NEC, surgical NEC, or NEC associated death. Since no major changes occurred in our NICU practice in recent years, and the introduction of * L. reuteri * as routine prophylaxis was abrupt, we attributed the post-probiotic changes to the introduction of this new therapy. Rates of NEC were compared using Chi square analysis with Fisher exact t-test.

**Results:**

Medical records for 311 neonates were reviewed, 232 before- and 79 after-introduction of * L. reuteri * prophylaxis. The incidence of NEC was significantly lower in the neonates who received * L. reuteri * (2 of 79 neonates [2.5%] versus 35 of 232 untreated neonates [15.1%]). Rates of late-onset gram-negative or fungal infections (22.8 versus 31%) were not statistically different between treated and untreated groups. No adverse events related to use of * L reuteri * were noted.

**Conclusions:**

Prophylactic initiation of * L. reuteri * as a probiotic for prevention of necrotizing enterocolitis resulted in a statistically significant benefit, with avoidance of 1 NEC case for every 8 patients given prophylaxis.

## Background

Probiotics are live microorganisms that provide beneficial effects when administered to humans. Use of probiotics in the neonatal intensive care unit (NICU) has been associated with decreased mortality and decreased severity and/or incidence of necrotizing enterocolitis (NEC) [1-5]. NEC is viewed a multifactorial disease with inflammation, destruction and bacterial invasion of the gut wall. Proponents of probiotic use argue that control of the bacterial flora in the gut can markedly affect NEC risk. Meta-analyses have provided further support for the value of these products [6,7]. However, other authors caution against routine use of probiotics, citing concerns with product quality and possible adverse effects [8,9]. Also questions remain about which bacterial strain(s) would be most beneficial. This places each NICU in the position of deciding whether they deem the evidence sufficient to selectively or routinely utilize probiotics in their practice. If they elect to employ probiotics as a NEC prevention strategy, each NICU must also decide which probiotic product to use. Since the bulk of the evidence focuses on using probiotics in neonates born < 1,500 grams [6], in our NICU these patients have become the target population for routinely initiating probiotic prophylaxis with the product * Lactobacillus reuteri * (* L. reuteri *) DSM 17938, available under the brand name BioGaia®(BioGaia, Sweden). Further reference to * Lactobacillus reuteri * in this paper specifically refers to this specific strain of * L. Reuteri * DSM 17938. This probiotic was initially selected because of relative ease of administration through nasogastric tubes without clogging the tube, compared to other products during our own in vitro experiments.

In this sequential analysis, our focus is to examine the potential benefits of * L. reuteri * in the highest risk group, neonates born ≤ 1,000 grams. This population is typically reported to have NEC rates of 10% [10].

## Methods

### Data collection

This study represents a retrospective chart review comparing the rates of NEC in neonates before introduction of * L. reuteri * (January 2004 – June 2009), with routine use of * L. reuteri * in neonates ≤ 1,500 gram birth weight (July 2009 – April 2011). The use of L. reuteri as a standard medicine in the <1500 gram infants in our NICU was a unanimous decision of the neonatologists and medical team at our institution, based on the substantial reduction in NEC rates seen in other studies using probiotics in this manner [3-5]. As is our practice when implementing new treatment strategies, we sought to evaluate the impact of the change to prophylactic probiotics, implemented in July 2009. The study to retrospectively review charts and measure outcomes was approved by the Institutional Review Board and exempted from needing informed consent. The study focuses on extremely low birth weight (ELBW) neonates born ≤ 1,000 grams, which is the highest at-risk group for NEC [10]. Medical records for all neonates in our NICU are routinely maintained in a unit-specific standardized database, NICU3®, which was designed and maintained by one of the authors (MS). Patient data is also categorized for reporting our NICU outcomes as part of the Vermont Oxford reporting process. The hospital electronic patient record was also used to collect study data.

Neonates were only excluded from the analysis if they died within the first week of life, as it was determined that these patients would not have had the opportunity to benefit from probiotic intervention. Data collected for this study included: gender, gestational age (determined by Ballard), birth weight, 5-minute Apgar score, growth restriction, any antenatal corticosteroid administration, method of delivery, maternal diagnosis of preeclampsia or chorioamnionitis and diagnosis of NEC or late-onset (after 7 days postnatal age) gram-negative bacteria or fungal infection. NEC was categorized as medical (Bell stage II NEC), surgical (Bell stage ≥3 NEC) or causing death (all associated with Bell stage ≥3 NEC) [11,12]. Neonates diagnosed with NEC were categorized into only one of the three groups (medical NEC, surgical NEC or death attributed to NEC), despite the patients who died all having surgical NEC as well.

Although the actual rates of breast feeding could not be reliably collected, our sense was that breast feeding practices were not overtly different during the period studied in this analysis.

### Probiotic administration, dosing and product selection

Starting in 2009, a probiotic in the form of * L. reuteri *, available under the trade name BioGaia® (BioGaia Inc., Lund, Sweden) was administered to preterm neonates. Beginning in July 2009, * L. reuteri * was administered to neonates once they crossed an empiric threshold for risk of developing NEC. Thus, initiation of BioGaia® was often beyond the first 1–2 weeks of life. In 2010, this practice was changed to empirically give BioGaia® to all neonates born ≤ 1,500 grams once feedings were started, almost exclusively within the first week of life, and continue administration until hospital discharge, as we have had NEC occur in this population as late as 40 weeks post-conception age.

According to the manufacturer of BioGaia®, for term neonates, the recommended daily dose is five drops (approximately 0.18 mL given the oil-based nature of the product drops). Five drops administers 100 million live active * L. reuteri * cells. In our preterm neonates, we opted to administer 0.1 mL as our standard dose. BioGaia® doses were prepared in the pharmacy under sterile conditions and refrigerated until administration the same day. Administration was required within six hours of removal from the refrigerator, per manufacturer recommendation.

We selected BioGaia® as our probiotic because the manufacturer has demonstrated careful quality control of the probiotic colonies delivered with their product. The manufacturer quality control process is performed at temperatures of 5, 25, and 30°C for 24 months, the listed shelf life of the product. At each of these temperatures, 5 drops of BioGaia®, is guaranteed to deliver 1x10^8^ colony forming units of L. reuteri (personal communication, Eamonn Connolly PhD, Senior Vice President Research, BioGaia AB, Stockholm, Sweden).

Additionally, other products examined in vitro, routinely clogged the orogastric tubes used in our neonates, and this was not encountered with BioGaia®.

### Statistics

Statistical approaches used to examine this data include unpaired * t- *test to examine differences in the demographic data collected before and after institution of BioGaia®. Statistically significant differences required * p * < 0.05 between groups. Comparisons of the incidence of NEC and late-onset infection for neonates not given probiotic prophylaxis (January 2004 – June 2009) and those routinely given prophylaxis with * L. reuteri * upon initiation of feedings (July 2009 – April 2011), utilized chi square analysis with Fisher exact * t- *test. Data is presented in yearly epochs to demonstrate the variability of NEC and late-onset sepsis in this population. Because the numbers of events are too small in yearly epochs to detect even large effects, no statistical analysis is applied to these data. We targeted a minimum of 80 neonates in the pre- and post- Lactobacillus reuteri for a p < 0.05 and a power of 0.8, assuming a reduction of NEC from 15% to 2.5%.

## Results

Patient demographics for included neonates born ≤ 1,000 grams are listed across years in Table 1 (n = 311). Rates of chorioamnionitis and small for gestational age neonates were the only variables found to be statistically different between the pre- and post- routine * L. reuteri * prophylaxis groups. Differences in NEC rates for SGA neonates and non-SGA neonates (7.4 versus 13.8%, * p * = 0.129) and for neonates of mothers diagnosed with chorioamnionitis and those not diagnosed with chorioamnionitis (15.1 versus 10.8%, * p * = 0.295) were not statistically significant.

**Table 1:** Patient characteristics

**Year**	**No BioGaia 2004 – 2009 (n = 232)**	**BioGaia 2009 – 2011 (n = 79)**
Male, %	48	49
GA (weeks), mean [SD]	26 [2]	26 [2]
BW (grams), mean [SD]	754 [151]	743 [175]
5-min Apgar, median [range]	7 [0–9]	7 [1-9]
Preeclampsia, %	26	25
Antenatal Steroids, %	95	95
Chorioamnionitis, %	53	36*
Cesarean Section, %	72	78
SGA, %	25	44*

Legend: GA, gestational age; SD, standard deviation; BW, birth weight; SGA, small for gestational age.

*p < 0.05.

The incidence of NEC and late-onset gram-negative bacteria or fungal infection in yearly epochs is summarized in Table 2. Comparison of events for the combined years before introduction of * L. reuteri * (January 2004 – June 2009), with the years of * L. reuteri * prophylaxis (July 2009 – April 2011), showed statistically significant lower rates of NEC (15.1 (35 of 232) versus 2.5 (2 of 79) %, p = 0.0475) after routine * L. reuteri * prophylaxis was started. A general decline in late-onset gram-negative bacterial or fungal infections was observed (31 (72 of 232) versus 22.8 (19 of 79) %), but was not statistically significant (p = 0.1112). The differences seen result in a number needed to treat (NNT) of 8 to prevent one case of NEC. Further, the lack of statistically significant benefit for prevention of gram-negative or fungal infections needs further study, since the current lack of statistical significance may miss a number needed to treat benefit as low as 12 to prevent one neonate from getting a gram-negative or fungal infection.

**Table 2:** Incidence of NEC and late-onset gram-negative or fungal positive cultures in ELBW infants from 2004 to 2011

**Year**	**2004 (n = 53)**	**2005 (n = 25)**	**2006 (n = 48)**	**2007 (n = 44)**	**2008 (n = 39)**	**2009**		**2010 (n = 36) #**	**2011 (n = 17) #**
						**n = 23**	**n = 26 #**		
Med NEC, No. (%)	4 (7.5)	2 (8)	4 (8.3)	1 (2.3)	4 (10.3)	1 (2)	0	0	0
Surg NEC, No. (%)	3 (5.7)	1 (4)	3 (6.3)	1 (2.3)	3 (7.7)	1 (2)	0	2 (5.6)	0
NEC-related death, No. (%)	2 (3.8)	1 (4)	1 (2.1)	1 (2.3)	1 (2.6)	1 (2)	0	0	0
Total NEC, %	17	16	16.7	6.8	20.5	6.1	0	5.6	0
Infection, No. (%)	16 (30.2)	4 (16)	20 (42.7)	17 (38.6)	13 (33.3)	2 (8.7)	6 (23)	7 (19.4)	6 (35.3)

# Patients treated routinely with prophylactic *Lactobacillus reuteri* DSM 17938.

The pattern of NEC prevention may also be important. As noted in Table 2, the rates of medical NEC and possibly fatal NEC appear more benefited than surgical NEC, although the numbers are too small to state this with confidence. The NEC cases associated with only medical treatment (antibiotics and restriction of oral feedings for at least two weeks) occurred in 16 (6.9%) neonates before instituting * L. reuteri * and none after instituting routine * L. reuteri * prophylaxis. This difference is statistically significant (* p * = 0.0143). Additionally, the combined effect of surgical NEC and fatal NEC (which are surgical cases), were reduced from pre- to post- introduction of * L. reuteri * (8.2 versus 2.5%). This difference is not statistically significant (* p * = 0.1774).

No adverse events or infections related to * L. reuteri * administration were noted in any of the infants included in the analysis.

## Discussion

Recent data from the Cochrane group have supported routine use of probiotics for prevention of severe NEC and all cause mortality in premature neonates born ≤ 1,500 grams [6]. While this population does not exclude infants born ≤ 1,000 grams, those authors caution against routine use of prophylactic probiotics in this high risk group until more data is published on their benefits and potential adverse effects [6]. In our NICU, the team elected to implement probiotics as a NEC prevention strategy. As part of our quality improvement initiative we elected to measure the impact of this strategy in the subpopulation of neonates born ≤ 1,000 grams who are at highest risk for developing NEC.^10^ We found this to be an effective strategy in contributing to reduced NEC rates in our ELBW population (15.1% to 2.5%). This effect is larger than seen in the studies for neonates with birth weight below 1500 grams, reported in the meta-analysis by Deshpande et al. [7] as decreasing from 6.56% in controls to 2.37% in probiotic-treated patients.

More experience with probiotic use in neonates born ≤ 1,000 grams is starting to be published. The first study exclusively evaluating probiotics in ELBW infants [13] did not find a difference in NEC rates, 4/51 (7.8%) in controls versus 3/50 (6%) in those receiving probiotics. This is likely in part due to inadequate power; however, their results do support safety of administering 500 million colony forming units (CFU) of * Lactobacillus GG * and 500 million CFU * Bifidobacterium infantis * once daily to their ELBW neonates. One caveat is that our experience with a lactobacillus GG product during our in vitro experiments was that it frequently clogged the nasogastric tube and would thus likely fail to be administered in a reliable manner. This could positively affect safety and negatively affect benefit results in a study. As previously stated, the larger published probiotic trials in neonates born ≤ 1,500 grams did not exclude neonates born ≤ 1,000 grams. In Lin and colleagues 2008 multicenter study evaluating * Lactobacillus acidophilus * and * Bifidobactrium bifidum *, presentation of their results by birth weight allows the reader to evaluate NEC rates in neonates born ≤ 1,000 grams, even though the study is not exclusive to this population. Their NEC rates in neonates born ≤ 1,000 grams were reduced from 7/79 (8.9%) in the control group to 4/102 (3.9%) in the group that received * L. acidophilus * and * B. bifidum * (NNT = 20, * p * =0.2245).^3^ The rate of NEC in our pre-* L. reuteri * control group was highly variable, ranging from 6.1% to 20.5%, and mostly ranging from 16% to 20%, with the average for the total population being 15.1%. This is higher than rates seen in other studies reporting neonates below 1000 gram birth weight, which are typically around 10% [14,15] and was a motivation to seek interventions that could improve the results. The dramatic improvement following introduction of * L. reuteri * lowered NEC rates below those seen in any NICU reports. In this particularly high-risk population, our progress is not likely to be a coincidence since the cycle of change has now affected 3 different epochs in our longitudinal study (Table 2).

Another related question is whether practice changes might account for the improved NEC rates. Against this is that rates of NEC were similar until * L. reuteri * was introduced and then remained similarly reduced after * L. reuteri * was made standard practice. Since our group tends to have similar practices and strives for continuity between attending physicians, and the same physician group have all been together throughout this study we cannot identify other practice variables that caused the changed NEC rates. We also did not make any other practice changes starting in 2009, such as increased breast milk usage or a different feeding strategy that we could identify to account for this improved NEC rate.

Neurodevelopmental consequences (cerebral palsy, cognitive impairment, visual impairment, hearing impairment) are of major concern for infants who survive NEC [16]. Infants born ≤ 1000 grams that develop NEC requiring surgical intervention have much poorer neurodevelopmental outcomes than those with medical NEC or no NEC [17]. In our analysis from July 2009 through April 2011, elimination of medical NEC and NEC-related death in ELBW neonates receiving * L. reuteri * prophylaxis is remarkable.

Probiotics have been shown to be a barrier to bacterial translocation and competitively exclude potential pathogens. Given these properties, one would conclude that probiotics should also play a role in reducing rates of infection. However, this has not been demonstrated in a controlled setting. Consistent with published literature [3,4,13,18,19] we did not find a significant reduction in incidence of culture-positive late-onset bacterial or fungal infection (31 versus 22.8%). Perhaps, as other authors have suggested, we have yet to understand the optimal probiotic product, dose and duration to reach this infection prevention threshold.

We chose to dose our preterm neonates daily with 0.1 mL of * L. reuteri *. This dose was a conservative reduction of what the manufacturer recommends for term infants. After two years of using this product, we wanted to confirm that the product was colonizing the infant gut in effective amounts. In 2011, we collected stool samples at random from 7 infants who had been receiving * L. reuteri * for varying lengths of time (12 – 101 days) and found that, while all 7 had some level of fecal colonization, 3 of the 7 infants did not have optimum fecal colonization (10^6^ to 10^7^ * L. reuteri * CFU/g stool). As a result, our NICU decided to increase our standard prophylaxis dose from 0.1 mL to 0.2 mL daily in order to more accurately administer the recommended 100 million live, active * L. reuteri * cells. Outcomes from this adjusted dose are currently being collected and are not reported in the present study.

One of the major criticisms surrounding probiotic use focuses on generalization of results to all probiotics strains. Therefore, it cannot be assumed that our results obtained with * L. reuteri * DSM 17938 can be extrapolated to other probiotic products. Each individual preparation must be analyzed separately in the context of other successful NEC prevention strategies.

Although we do not completely understand the pathogenesis of NEC, it is recognized that NEC involves multiple contributing factors unique to preterm infants. Probiotics have been shown to assist in decreasing NEC rates, but given the multi-factorial nature of the disease, we would expect prevention to ultimately require a multi-modal strategy as well.

## Conclusions

Prophylactic initiation of *Lactobacillus reuteri* as a probiotic for prevention of NEC resulted in statistically significant reduction in NEC. Our results support that 1 NEC case is avoided for every 8 cases given * L. reuteri * prophylaxis. The benefits were especially focused on the subsets of medical NEC and death related to NEC in infants born ≤ 1000 grams. These results suggest a significant benefit to * L. reuteri * prophylaxis in this subset of neonates.

## Abbreviations

NICU: Neonatal intensive care unit; NEC: Necrotizing enterocolitis; NNT: Number needed to treat; CFU: Colony forming units; ELBW: Extremely low birth weight.

## Competing interests

The study was partially funded through a grant from BioGaia Inc. The author(s) declare that theyhave no competing interests.

## Authors’ contributions

The authors on this paper all participated in design and implementation of the *L. reuteri* prophylaxis protocol. The neonatologists were responsible for the final decision regarding NEC classification. All authors read, critiqued and approved the manuscript revisions as well as the final version of the manuscript. Also, all authors participated in a session to discuss the results and consider strategies for analysis and interpretation of the data before the final data analysis was performed and the manuscript written. The main database used to capture patient information was designed by MS. Data collection was primarily performed by CH in her role as Neonatal Pharmacotherapy Resident at Women’s Hospital, and monitored by the co-principle investigators MAVTD and PG. Statistical analysis was performed by PG. All authors read and approved the final manuscript.

## Pre-publication history

The pre-publication history for this paper can be accessed here:

http://www.biomedcentral.com/1471-2431/12/142/prepub
